# Synthetic Pentatricopeptide Repeat Proteins: Building a Toolkit for Precise RNA Control

**DOI:** 10.3390/ijms262412033

**Published:** 2025-12-14

**Authors:** Jose M. Lombana, Maureen R. Hanson, Stephane Bentolila

**Affiliations:** Molecular Biology Department, Cornell University, Ithaca, NY 14853, USA; jml498@cornell.edu (J.M.L.); sb46@cornell.edu (S.B.)

**Keywords:** pentatricopeptide repeat (PPR) proteins, programmable RNA recognition, synthetic biology, RNA engineering, RNA editing

## Abstract

In plants, cytidine-to-uridine (C-to-U) and uridine-to-cytidine (U-to-C) editing events are directed by pentatricopeptide repeat (PPR) proteins, modular RNA-binding factors that recognize their RNA targets through a predictable amino acid–nucleotide recognition code. Deciphering this code has enabled the rational design of synthetic PPR (synPPR) proteins with programmable RNA-binding specificity and robust stability in heterologous systems. Recent advances have extended these synthetic scaffolds to active RNA editors by fusing them to catalytically competent DYW deaminase domains, generating customizable enzymes capable of precise base conversion in bacteria, plants, and even human cells. This review summarizes current understanding of the structural and mechanistic principles underlying PPR-mediated RNA editing and highlights recent progress in the design and application of synPPR proteins. We discuss how synthetic PPR proteins have been used as programmable RNA stabilizers, translational regulators, and targeted C-to-U or U-to-C editors, as well as their emerging therapeutic potential in RNA-mediated diseases. The development of compact, cofactor-independent editors derived from early-diverging plant lineages further expands the versatility of this platform. Together, these efforts establish synthetic PPR proteins as a powerful and flexible class of RNA engineering tools with applications spanning basic research, biotechnology, and biomedicine. Continued refinement of targeting specificity, catalytic efficiency, and effector modularity will propel PPR-based editors toward broader use in synthetic biology and therapeutic RNA modulation.

## 1. Introduction

**Figure 1:**
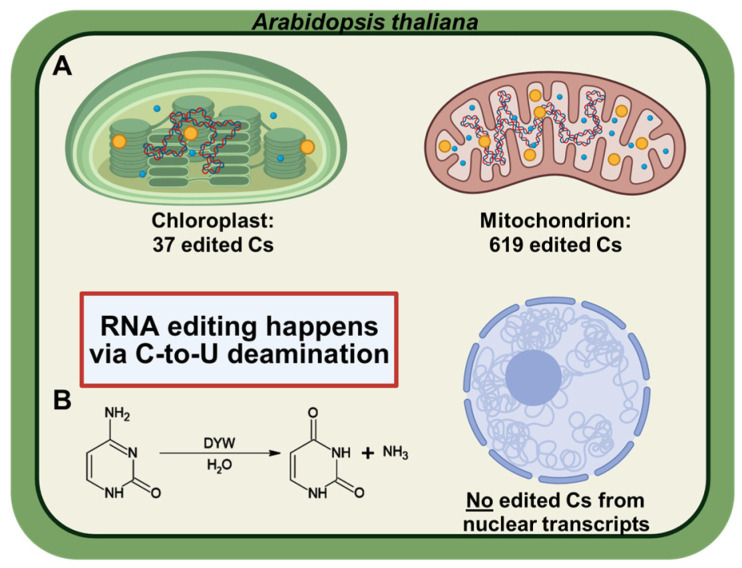
RNA editing in *Arabidopsis thaliana*. (**A**) Schematic representation of an *A. thaliana* cell highlighting organellar RNA editing. In plants, RNA editing is restricted to organellar transcripts (mitochondrial and chloroplast mRNAs); no editing activity has been reported in nuclear transcripts. (**B**) Mechanism of C-to-U RNA editing in plants. The conversion of cytidine to uridine is catalyzed via hydrolytic deamination.

RNA editing is a process that alters genetic information at specific sites on RNA molecules. Editing has been described in a wide range of organisms from viruses to animals and plants. Several systems involving unrelated mechanisms seem to have arisen separately during evolution (for review, see [1]). Plants extensively edit organellar transcripts, cytidine-to-uridine (C-to-U) conversions in their plastids and mitochondria [2,3,4,5,6] (Figure 1) but also U-to-C conversions in several early-diverging “hyper-editing” lineages such as hornworts, lycophytes, and ferns [7,8]. These edits often restore evolutionarily conserved codons, including start or defective codons, thereby rescuing protein function [9,10]. Failure of editing frequently causes loss of function of the targeted genes in vivo [11,12,13], providing an additional post-transcriptional layer that constrains organellar gene expression [14,15].

RNA editing is just one of the many steps of RNA maturation and metabolism in plant organelles that rely on the function of the pentatricopeptide repeat (PPR) protein family, which is by far the largest family of plant RNA-binding proteins (RBPs) [16]. The PPR protein family possesses several attributes that make it a promising resource for the development of synthetic RBPs as tools to manipulate RNA. PPR proteins display modular structure like other known RBP families such as the Pumilio/fem-3 mRNA-binding factor (PUF) [17], mitochondrial transcription termination factor (mTERF) [18], octatricopeptide repeat (OPR) [19], heptatricopeptide repeat (HPR) [20] and half-a-tetratricopeptide repeat (HAT) proteins [21]. In addition, PPR proteins tolerate C-terminal domains such as the small MutS-related (SMR) domain carrying endonuclease activity [22]. The distribution across all plant lineages associated with vast diversity and available sequences databases of PPR proteins also reinforce their superiority as the best candidates to provide scaffolds for synthetic RNA editors. Finally, the deciphering of the recognition code between the PPR protein and its RNA target has allowed the design of synthetic PPR proteins with predictable RNA-binding capabilities [23].

The goal of this review is to provide an updated overview of synthetic PPR editors, with a focus on their design principles, uses in bacterial, plant, and human systems.

## 2. Mechanisms of PPR-Mediated RNA Editing

### 2.1. PPR Proteins

PPR proteins form a huge family in plants that comprise, for example, 450 members in Arabidopsis and 477 in rice [24], most of which are predicted to be targeted to either mitochondria or plastids [25]. The PPR genes were first identified in the genome of *A. thaliana* as encoding proteins characterized by degenerate motifs, which are around 35 aa long, related to the tetracopeptide repeat (TPR), and present in 2–30 tandem repeats [26]. Each motif is folding into a pair of antiparallel alpha helices; the tandem array of PPR motifs produces a super helix whose central groove forms a binding surface for the RNA ligand [16]. While most PPR proteins bind to mRNA, Arabidopsis PRORP1, which contains 3 PPR motifs, binds to tRNA precursors and performs their endonucleolytic maturation [27]. Yeast PPR protein DMR1 binds to the mitochondrial 15S rRNA and is required for its maintenance [28].

**Figure 2:**
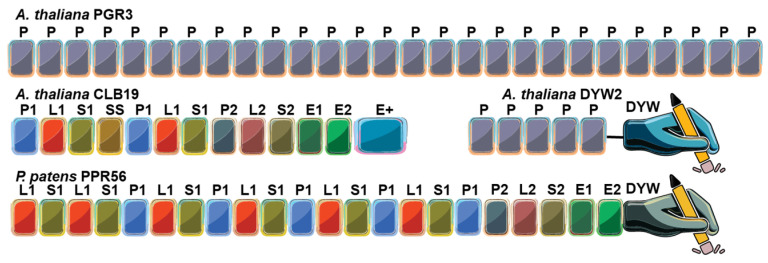
Overall domain architecture of representative endogenous pentatricopeptide repeat (PPR) proteins. The P-type PPR protein PGR3 (*Arabidopsis thaliana*) binds the 5′ untranslated region (UTR) of *petL* mRNA, stabilizing the transcript and promoting translation of the downstream gene product. The PLS-type PPR protein CLB19 (*Arabidopsis thaliana*), which recognizes specific chloroplast RNA editing sites, functions together with the DYW2 protein that provides the cytidine deaminase (C-to-U) activity. CLB19 serves as the sequence-specific RNA recognition factor, while DYW2 contributes the catalytic domain. The PLS-type PPR protein PPR56 from the moss *Physcomitrium patens* contains a C-terminus DYW domain and catalyzes C-to-U RNA editing autonomously. Key to domains: P, P-type PPR motif; P1, P2, L1, L2, S1, S2, variants of the PLS-type PPRs; E1, E2, Extended motifs; E+, Extended plus domain (degenerate/half-deaminase); DYW, cytidine deaminase domain.

Plant PPR proteins can be separated into two major classes based on the nature of their PPR motifs, the P-class and PLS-class (Figure 2). The P class proteins contain only canonical P motifs of 35 aa, are found throughout the plant kingdom and in most eukaryotes, and often act in RNA stabilization, splicing, or translation [25,29,30]. Sometimes additional domains are added at the C-terminus that confer specific functionality such as the small MutS-related (SMR) that is involved in RNA cleavage [31] or the restorer of-fertility C-terminal domain [32]. The PLS-class proteins contain triplet motif repeats made of P, L (long), and S (short) motifs, where L motifs are 35–36 amino acids and S motifs are 31 amino acids, both variants of the standard PPR motif. PLS proteins often contain at their C-terminus two PPR-like motifs E1 (34 aa) and E2 (34 aa), which often precede a 135–136 aa DYW domain responsible for catalyzing C-to-U RNA editing [25,33]. PLS-class PPR proteins are almost exclusively involved in RNA editing [34,35,36]. A variant of the DYW domain that is present in hornworts, lycophytes and ferns [8] and denoted DYW:KP has been demonstrated to catalyze the ‘reverse’ U-to-C editing [37].

### 2.2. The PPR Code

**Figure 3:**
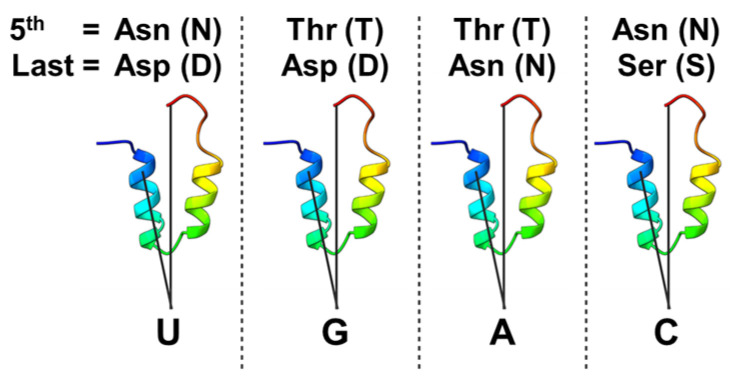
The pentatricopeptide repeat (PPR) code. A representation of the amino acid combinations at the 5th and last positions of each PPR that determines nucleotide recognition specificity. The lower panel shows a predicted structure of a single PPR, colored from the N-terminus (blue) to the C-terminus (red). The connecting lines indicate the positions of the 5th and last amino acid residues within each repeat that form the basis of the so-called PPR code. U (uridine), G (guanine), A (adenine), C (cytosine).

The PPR-PLS protein is the principal specificity factor of plant organellar RNA editing [11,13,38]. The PPR tract provides RNA specificity by binding to bases upstream of the edited C [9,14,39]. The mode of recognition of the target RNA by the PPR proteins was elucidated through the study of particular PPR proteins and their RNA ligands. First, the one repeat: one nucleotide recognition mode was supported by correlations between the number of PPR motifs and the number of ribonucleotides in the binding sites [30]. Patterns of diversifying selection in the fertility restorer (Rf) genes, all encoding PPR-P proteins, hinted at which residues were important for forming base-specific contacts to the RNA ligand [40]. Finally, correlations between these residues and aligned RNA bases were first described in a study of the maize chloroplast P-class PPR protein PPR10, which binds as a monomer to 5′ untranslated regions of the plastid *psaJ* transcript [41]. The specificity of the ribonucleotide bound is conveyed in each PPR motif by two key amino acids at the fifth and last position. Asparagine at the fifth position correlates strongly with pyrimidine at the corresponding position in the RNA, whereas serine or threonine correlates with purine (Figure 3). At the last residue position aspartate correlates with uridine or guanosine, whereas asparagine correlates with cytidine or adenosine. Similar results were obtained for P and S PPR motifs by aligning RNA editing PPR-PLS factors with the *cis* elements found upstream of their RNA targets [42]. Crystal structure of PPR10 in a RNA-bound state confirmed the molecular recognition of A, G and U by PPR motifs via hydrogen bonds [39]. This combinatorial code has been used to predict the targets of natural editing factors [42], to modify the specificity of PPR proteins both in vivo and in vitro [43] and to design de novo synthetic PPR editors aimed at chosen target sites [44].

### 2.3. The DYW Deaminase Domain

**Figure 4:**
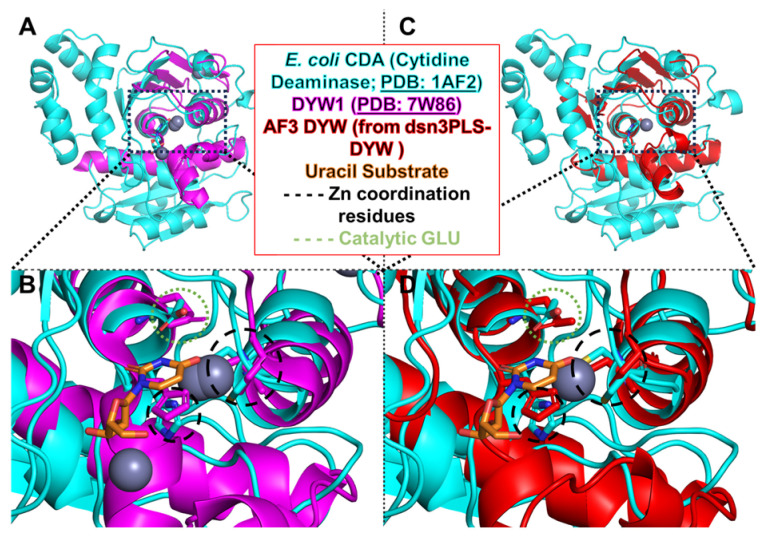
Comparison of plant DYW deaminases with the ancestral bacterial cytidine deaminase (CDA) from *Escherichia coli*. Superimposed structures reveal that plant DYW deaminases share striking structural similarities, specifically around their catalytic site with the bacterial CDA (PDB: 1AF2), despite differences in the overall fold. (**A**) Crystal structure of the *A thaliana* DYW1 protein (PDB: 7W86) aligned with CDA, highlighting the conserved catalytic zinc-binding core. (**B**) Closeup of the matching central alpha helices and the conserved catalytic zinc-binding core. (**C**) AlphaFold3-predicted structure of the DYW domain from the synthetic editor dsn3PLS-DYW, shown superimposed with CDA, (**D**) further illustrating conservation of the active-site geometry including the catalytic glutamate residue.

The DYW domain, named after the conserved amino acids aspartate (D), tyrosine (Y) and tryptophan (W), frequently occurs at the end of the PPR-PLS editing factors (Figure 4) [25]. This domain has long been thought to carry the deaminase activity because of its phylogenic co-occurrence with plant taxa that show RNA editing [45]. In addition, the DYW domain harbors a conserved cytidine/deoxycytidylate deaminase motif (that is HxERx24CxxC; InterPro IPR002125). The apparent paradox about the DYW motif providing the deaminase activity was the dispensable presence of this domain in many truncated PPR-PLS editing factors. This observation was later clarified by the recruitment in *trans* of DYW donor proteins by editing factors lacking the deaminase domain [46,47]. Experimental evidence for the deaminase activity of the DYW domain came from mutagenesis studies of conserved residues in the deaminase signature sequence. Mutation of the glutamate or any of the three zinc-coordinating residues in plant DYW editing factors completely abolishes editing at the sites under their control [48,49]. Strong evidence for the deaminase activity of the DYW domain came from the expression of a PPR-DYW protein from *P. patens* in bacteria along with its target sequence, which resulted in robust editing [33]. Ultimately the same purified, recombinant PPR-DYW exhibited robust editase activity on synthetic RNAs containing a cognate in vitro target, indicating that a PPR protein with a DYW domain is solely sufficient for catalyzing C-to-U RNA editing in vitro [50].

### 2.4. The Editosome

In early land plants such as the moss *P. patens*, the editosome, the molecular apparatus responsible for the ribonucleotide conversion, is very simple and made up of a single PPR-DYW protein. Individual moss PPR-DYW proteins are sufficient for C-to-U editing when expressed alone in heterologous systems, including *E. coli* and purified in vitro [33,50]. By contrast, in angiosperms, efficient editing occurs in multi-protein editosomes comprising the site-defining PPR proteins plus accessory factors. First evidence for the presence of other *trans* factors in addition to the PPR-PLS protein came from genetic studies where mutation in these accessory proteins abolishes editing at many organellar sites. The RIP/MORF family was the first family of accessory proteins to be described in *Arabidopsis thaliana* [51,52,53]. RIP1 (MORF8) broadly affects RNA editing as altered efficiency was detected for 14 chloroplasts targets in the *rip1* mutant while 108 mitochondrial targets exhibited major loss of editing [51]. The RIP family contains 10 members, among which five are localized to mitochondria, two reside within chloroplasts and two are dual localized to both organelles. MORF2 (RIP2) and MORF9 (RIP9) are involved in most editing events in chloroplasts, while MORF1 (RIP8) and MORF 3 (RIP3) act at more than 50 sites in mitochondria [52,53]. Several RIP/MORF proteins were shown to interact selectively with PPR-PLS editing factors by yeast two hybrid, bimolecular fluorescence complementation and pulldown assays [54]. The function of these accessory proteins was elucidated for at least one of its members, MORF9, by a co-crystal structural analysis with a synthetic PPR-PLS protein [55]. MORF9 binding to the synthetic protein primarily mediated by the L-type motif induces a significant conformational change, resulting in increased RNA-binding activity. Electromobility shift assays (EMSAs) demonstrated the increased affinity of the synthetic PPR-PLS protein when complexed to MORF9 for its RNA target [55].

The second non-PPR protein family to influence editing of many organellar sites is termed the Organelle RNA Recognition Motif (ORRM)-containing proteins. ORRM1, the founding member of this family, was identified because it contains a pair of truncated RIP domains (RIP-RIP) [56]. ORRM1 is an essential plastid editing factor; in Arabidopsis and maize mutants, RNA editing is impaired at particular sites, with an almost complete loss of editing for 12 sites in Arabidopsis and 9 sites in maize. In addition to its RIP-RIP domain, ORRM1 carries a RRM at its C terminus; transfection of *orrm1* mutant protoplasts demonstrated that the RRM is sufficient for the editing function of ORRM1 in vitro. Subsequently other members of this family were found to be involved in mitochondrial (ORRM2, ORRM3, ORRM4) [57,58] or plastid editing (ORRM6) [59]. The function of these accessory proteins in the editosome remains unclear; however, co-expression of ORRM1 with a synthetic editing factor in bacteria fully supports its role as an editing factor [60]. Furthermore, the same report demonstrated that ORRM1 and RIP2 (MORF2) or RIP9 (MORF9) act additively to contribute to the editing of the RNA target by the synthetic factor suggesting an independent function in editing of ORRM and RIP proteins.

**Figure 5:**
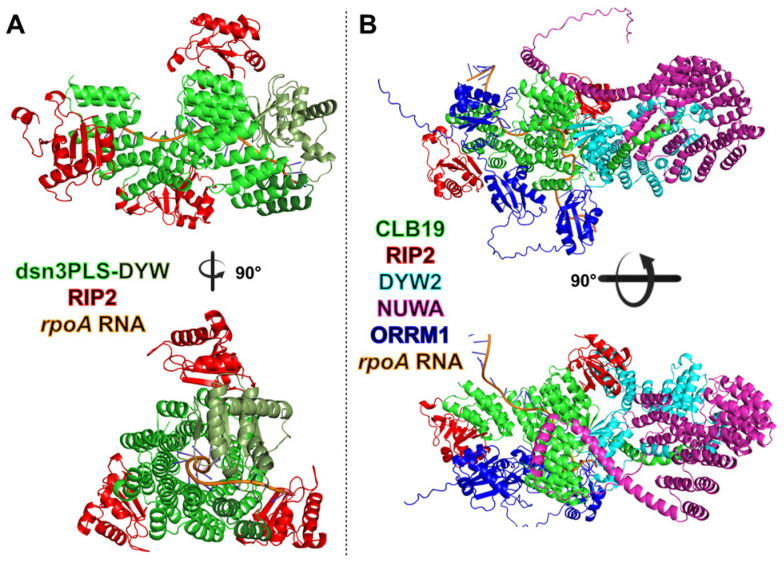
Predicted minimal synthetic editosome compared with the endogenous *A. thaliana* plastid *rpoA* editosome. (**A**) AlphaFold3-predicted structure of the minimal synthetic editosome composed of the engineered PPR protein dsn3PLS-DYW (green) in complex with three RIP2 accessory proteins (red) bound to the *rpoA*-C200 plastid RNA target. The DYW deaminase domain of dsn3PLS-DYW is shown in dark green. (**B**) AlphaFold3-predicted structure of the endogenous *A. thaliana rpoA*-C200 plastid editosome. The PLS-type PPR protein CLB19 (E+) (green) interacts with two RIP2 proteins (red), the catalytic DYW2 protein (cyan), and is further stabilized by the P-type PPR protein NUWA (purple). ORRM1 (blue) associates with the RNA segment emerging from DYW2 and engages the PPR tract through its two RIP domains.

Additional non-PPR editing factors in plant organelles include individual proteins like the protoporphyrinogen IX oxidase 1 (PPO1) protein [61], the RNA helicase ISE2 [62], and OZ1 (Organelle Zinc finger 1) [63]. PPO1 is an enzyme in the tetrapyrrole biosynthetic pathway and its involvement in editing was rather surprising. The *ppo1* mutant plants show a reduction in editing extent at several plastid sites but a total suppression for only the *ndhD*-2 site, where ACG is partially edited (50%) into the translation start codon AUG in the wild-type plant. PPO1 interacts with several plastid MORFs but not with PPR-PLS editing factors, suggesting that this protein controls the stability of MORF proteins through physical interaction. ISE2 functions in multiple aspects of chloroplast RNA processing and metabolism including C-to-U RNA editing and ribosome biogenesis. OZ1 was found by co-immunoprecipitation of the chloroplast editing factor ORRM1 and is essential for chloroplast editing at 14 sites. The OZ family comprises four members for which only OZ1 has been shown to be an editing factor. OZ2, which is mitochondrial, is a splicing factor [64]. The function of the remaining two OZ proteins remains to be determined. Pulldown experiments in maize using an antibody against RIP9 identified with high confidence several PPR editing factors with the non-PPR proteins RIP9, RIP2, RIP1, ORRM1, OZ1 and ISE2, further supporting their presence in plastid editosomes [65]. In addition to these non-PPR accessory components of the editosome, NUWA is a P-class PPR protein identified as a core member of some editosomes [47,66]. NUWA was isolated as an interacting partner of mitochondrial factor SLO2 or chloroplast factor CLB19, both of which are E+ subclass PPR-RNA editing factors devoid of a DYW domain. NUWA assists in the interaction between SLO2 and DYW2, a short PPR-PLS-DYW protein that brings a functional catalytic DYW domain. This bridging function of NUWA is hypothesized to occur with other PPR-PLS-E+ proteins. Figure 5 shows an AlphaFold prediction of a synthetic and a natural editosome.

As a result of these investigations on the composition of angiosperm editosomes, it appears that these protein complexes are highly diverse not only with regard to the PPR proteins they contain but also in their non-PPR components [67].

## 3. Synthetic PPR Proteins

Natural PPR proteins, while essential for organellar RNA processing, are often difficult to express and purify outside organelles, motivating consensus-designed scaffolds with improved stability and solubility. By utilizing large multiple sequence alignments of natural PPR motifs, the most over-represented amino acids at each position can be determined, while amino acids at position 5 and the last position of the synthetic PPR motif can be chosen according to the PPR-RNA recognition code. Because of their enrichment over vast evolutionary timescales, these amino acids are predicted to be best suited to enhance that domain’s activity or stability.

### 3.1. Design of Synthetic PPR Proteins

**Figure 6:**
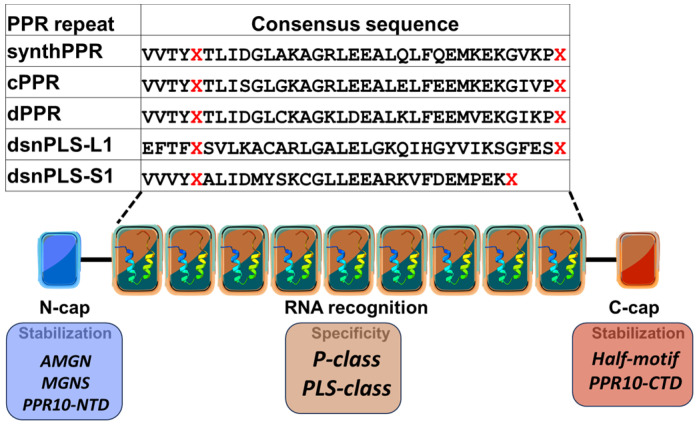
Consensus sequences of synthetic PPR protein repeats. For each repeat type, the **Xs** correspond to the fifth and last amino acids that determine nucleotide recognition. SynthPPR [68], cPPR [70], dPPR [55,69], dsnPLS-L1 [55] and dsnPLS-S1 [55] are consensus motifs developed from different databases.

The vast database available of PPR sequences allows the design of different synthetic PPR motifs, depending on the source of sequences chosen by the investigator. Gully et al. based their design on 2357 *A. thaliana* PPR motifs of exactly 35 amino acids [68], while other groups based their design on the consensus of all P-type PPR genes from *A. thaliana* [69] or the collection and curation of 23,916 P-class PPR sequences obtained from the UniProtKB database [70] (Figure 6). Another similarity in these different designs concerns the termini of the synthetic PPR protein where motifs were added to increase the stability and solubility. However, the nature of these motifs differed between the different designs. At the N terminus is added a short α helix stabilizing sequence (AMGN) in Gully et al.’s design, while a nucleating cap (MGNS) with high propensity to occur at this position or part of the N terminal domain of PPR10, a natural protein, were placed in designs by Coquille et al. and Shen et al., respectively [69,70]. A C-terminal solvating helix used previously in synthetic TPR proteins to prevent protein unfolding was added to the synthetic protein designed by Coquille et al. A different C-terminal stabilization domain is found in the form of a half of a PPR motif or part of the C terminal domain of PPR10 in Gully et al. and Shen et al., respectively. Other similarities relate to the departure from a strict consensus sequence dictated by concerns that certain consensus amino acids could affect the structure of the protein. For instance, cysteine is substituted with an alanine or with a glycine to reduce the potential for unwanted disulfide-bond formation [68,70]. As anticipated, the thermal stability and solubility of the synthetic PPR proteins were greatly improved compared to their natural counterparts [68,70]. These first synthetic proteins were shown to bind selectively and with high affinity to homopolymeric RNA probes composed of A, C, U according to the PPR code, T(S)N:A, NS:C, TD:G, ND:U (G was challenging to study because of the propensity of G tracts to form stable quadruplex structures) [69,70]. Coquille et al. were able to support the selectivity of TD for G by studying the affinity of a synthetic protein for a heteropolymeric RNA target containing a single guanine [70].

The design developed by Shen et al. [69] was further investigated in a study of RNA-binding specificity landscapes of synthetic PPR proteins [23]. In this study, the length of the synthetic proteins was extended from the original 10 PPR motifs to 11 and 14 motifs. The specificity of these proteins for the RNA ligands was confirmed to be accounted for by canonical code-based nucleotide recognition. An important outcome from this study shows that proteins with 11 and 14 repeats exhibit similar affinity for their intended targets but 14-repeats are more permissive for mismatches. This work suggests that there is some optimal PPR tract length of 10–11 PPR motifs that maximizes affinity for a specific RNA sequence while minimizing tolerance for mismatches.

The first designer PLS-type PPR protein, (PLS)3PPR, was designed by Yan et al. [55] and constituted by the repetition of 3 PLS units. The P-type motif was identical to the one designed by Shen et al. [69] while the alignment of 263 L-type motifs and 1117 S-type motifs from *A. thaliana* was used to derive the consensus motifs for L and S, respectively. The addition of motifs from PPR10 at the N and C-termini to optimize the solubility of the synthetic protein resembles the Shen et al. design but with a much smaller size. The crystal structures of the synthetic protein alone or in complex with MORF9 showed that the binding of MORF9 induces significant conformational changes to (PLS)3PPR increasing its RNA biding affinity.

### 3.2. Use of Synthetic PPR Proteins as Passive Binders to RNA (DNA) Targets

**Figure 7:**
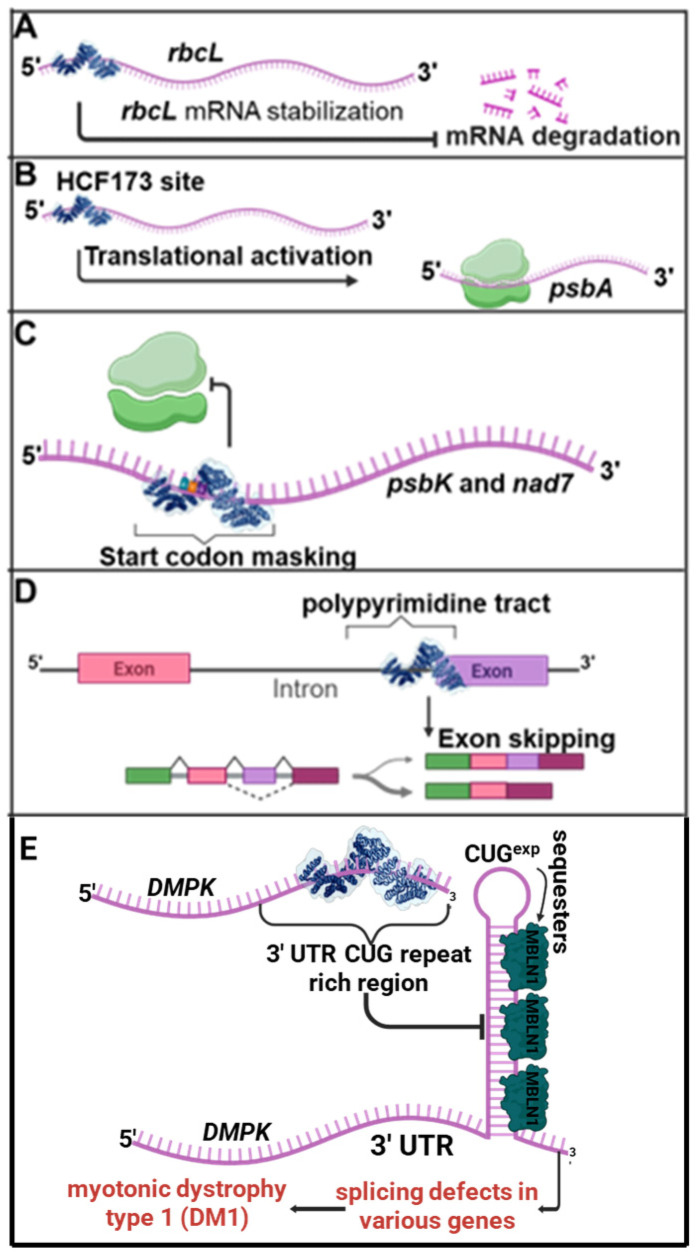
Synthetic PPR proteins used for translational control. Representative examples of synthetic pentatricopeptide repeat (synPPR) proteins engineered for non-editing functions in gene expression regulation. (**A**) A synPPR protein targeting the 5′ untranslated region (UTR) of the *rbcL* gene stabilizes the RNA by protecting it from 5′ exoribonuclease degradation. (**B**) A synPPR protein designed to bind the HCF173 recognition site in *A. thaliana hcf173* mutant plants promotes translational enhancement and partial phenotypic rescue in functional complementation assays. (**C**) A synPPR protein designed to bind and mask the start codon of the *psbK* and *nad7* genes inhibits ribosome binding and translation initiation. (**D**) A synPPR protein engineered to bind an intronic region inhibits splicing by preventing spliceosome formation. Targeted at specific polypyrimidine intronic sites, it promotes intron skipping. (**E**) A synPPR protein engineered to recognize and bind the CUG-expanded repeat region in the 3′ untranslated region (3′ UTR) of the *dystrophia myotonica protein kinase* (*DMPK*) mRNA disrupts the formation of aberrant RNA secondary structures. By preventing sequestration of splicing regulators such as Muscleblind-like 1 (MBNL1), this intervention alleviates mis-splicing events associated with myotonic dystrophy type 1 (DM1) in both mammalian cell culture and mouse models.

Passive binding of natural PPR proteins to RNA targets in vivo is sufficient to explain many of the ensuing effects on expression of the target transcript, such as RNA stability or translation activation [30,71]. Synthetic PPR proteins have been conceived to mimic these functions in plant organelles (Figure 7); among these proteins, several have used the design developed by Shen et al. [69,72,73,74]. The artificial PPR protein developed by Manavski et al. was designed to bind a sequence near the 5′ end of *rbcL* transcripts in Arabidopsis chloroplasts [72]. It was shown to substitute for the function of a natural PPR protein by stabilizing processed *rbcL* mRNA. In the work by Rojas et al., a synthetic PPR protein was engineered to substitute for HCF173, which is not a PPR protein but binds to the *psbA* 5′-untranslated region (UTR) [73]. HCF173 has been hypothesized to enhance translation by binding an RNA segment that would otherwise pair with and mask the ribosome binding region. The synthetic PPR was shown to bind to the intended 5′UTR site in vivo and to partially substitute for HCF173 by activating *psbA* translation.

The opposite goal of translation inhibition was pursued in another study by Manavski et al. [74], where synthetic proteins were engineered to specifically inhibit the translation of organellar mRNAs by masking their start codons. Two PPR proteins were designed to bind to the RNA area surrounding the start codon in the *psbK* plastid transcript and the mitochondrial *nad7* transcript. Both synthetic proteins were able to inhibit the translation of their targets with high specificity in vivo and resulted in the expected phenotypes corresponding to a pronounced decrease in PsbK or Nad7. This result represents an exciting way of manipulating gene expression in both organelles, but especially in mitochondria where there is no feasible method of transformation and laborious strategies have been developed to manipulate mitochondrial gene expression.

Another interesting investigation involving synthetic PPR pertains to the use of single molecule fluorescence energy transfer to study the mechanism of ssRNA binding to individual synthetic PPR proteins in real time [75]. This study demonstrated the reduced ability of a PPR protein to bind to sites containing secondary structures, conflicting with the proposed mechanism of translation upregulation. In this model, binding of the PPR protein to a secondary structure sequestering the ribosome binding site frees it and makes it accessible to the ribosome. However as noted by the authors, this role might be performed in vivo in collaboration with other proteins such as RNA helicases, chaperones or sRNAs. Importantly, this study shows that the synthetic PPR does not scan longer ssRNA oligonucleotides for the target sequence. This so-called random 3D diffusion may have important implications for the use of synthetic PPR proteins in heterologous cellular contexts different from the plant organelles. The complexity of the transcriptome in cytoplasmic settings might prevent the use of the synthetic PPR proteins unless the scanning speed of PPRs for their targets can be improved.

Alternative splicing in mammalian cells was achieved by designing PPR proteins targeting the *cis*-sequences in pre-mRNA preventing the binding of *trans*-splicing factors [76]. A bi-chromatic reporter gene expressing different fluorescence upon exon skipping demonstrated that synthetic PPR proteins could be used to control splicing in mammalian cells (Figure 7). The same strategy was used to control the splicing of an endogenous gene, *checkpoint kinase 1* (*CHK1*), that is a key regulator of the checkpoint signaling in both the unperturbed cell cycle and DNA damage response. Exon skipping of the *CHK1* pre-mRNA was successfully achieved in the same cells.

Recently a synthetic PPR protein was used to target a pathogenic RNA sequence in a mouse model of Myotonic Dystrophy type 1 (DM1) [77]. DM1 is a multisystem disorder caused by the expansion of a CTG-triplet repeat in the 3′ untranslated region of the *dystrophia myotonica protein kinase* (*DMPK*) gene. The toxic CUG repeat (CUG^exp^) RNA sequesters splicing factors, disrupting the normal splicing program that is essential for various cellular functions. A synthetic PPR protein designed to bind an hexamer of CUG repeats could ameliorate RNA toxicity induced by CUG^exp^ in cell models of DM1 (Figure 7). Furthermore, an adenovirus delivery of the synthetic PPR protein demonstrated long-term therapeutic effects on myotonia and restored splicing activity in a mouse model of DM1. It is the first example of a successful therapeutic use of synthetic PPR proteins and highlights the potential of these RNA-binding proteins in RNA-mediated disorders.

It is noteworthy that the synthetic P-type PPR proteins not only bind RNA molecules but also single stranded DNA (ssDNA) [78]. The same code and specificity as for RNA binding is responsible for interaction with the ssDNA. An engineered PPR protein was designed to target the telomeric ssDNA and blocked telomerase activity. This study demonstrated another interesting application of the synthetic PPR proteins.

### 3.3. The Synthetic PPR (synPPR) Editors

**Figure 8:**
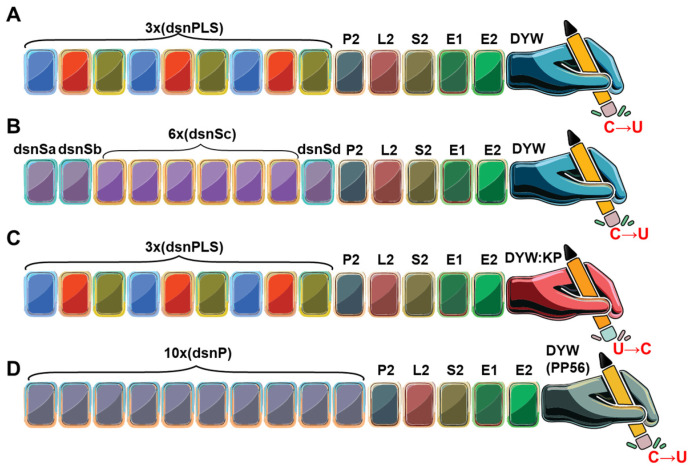
Overall architecture of representative synthetic PPR editors. (**A**) The dsn3PLS DYW protein, designed from angiosperm consensus sequences of P, L, and S repeats. (**B**) The 9S DYW synthetic PPR (synPPR) protein, constructed from consensus sequences of S-type abundant in non-vascular plants and in the Selaginella genus. This synPPR editor exhibits robust RNA editing activity without the requirement for accessory proteins.(**C**) The DYW:KP protein, assembled using the dsn3PLS PPR tract but containing a modified catalytic domain (DYW:KP) that catalyzes reverse RNA editing, converting uridine to cytidine (U to C). (**D**) The synthetic PPR editor dPPRe, constructed from consensus sequences of P-type PPRs fused with the DYW domain of the moss PPR56 protein.

The first synthetic PPR editor, dsn3PLS-DYW, was designed by Royan et al. with a motif arrangement (P1-L1-S1)3-P2-L2-S2-E1-E2-DYW (Figure 8) [79]. The design incorporated the most representative amino acids at each position in each motif based on 9730 PPR protein sequences from 38 different species, largely seed plant species, but also including *P. patens*, *Selaginella moellendorffii*, and *Picea abies*. At the N terminus was the same cap (MGNS) used in the design by Coquille et al. [70]. The amino acids at the 5th and last position in each motif of (P1-L1-S1)3-P2 were chosen according to the PPR code to bind the plastid *rpoA* target but not the *clpP1* target, both recognized in vivo by the natural PPR protein CLB19. Unlike CLB19, which lacks a DYW motif and relies on a *trans* DYW to edit its targets, dsn3PLS-DYW is designed to bind and edit the *rpoA* target. RNA electrophoretic assays demonstrated specific binding of the synthetic editor for its target that was greatly improved in the presence of the MORF9 accessory protein. The dependency of the synthetic editor on the presence of MORF9 was also observed for editing of the *rpoA* target in bacteria, which increases from about 10% when the synthetic factor was solely expressed to about 35% when it was co-expressed with either the co-factor MORF2 or MORF9. The synthetic factor was able to partially complement the editing defect of the *clb19* mutant plant to an editing extent of 45%. Off-target events were only detected in the chloroplast, not in bacteria, presumably because the expression vectors for the synthetic editor and the MORF proteins shared the same origin of replication. Hence the co-expression of a MORF protein reduced the expression of the synthetic factor by more than an order of magnitude. The drop in levels of the editor combined to high abundance of the *rpoA* target in the bacterial settings might have resulted in sequestering of a large fraction of the synthetic editor. Nevertheless, this study served as a successful pilot into the design and application of programmable RNA editing factors based on plant PPR proteins.

The same synthetic editor was used in a recent study to decipher the roles of the accessory proteins in the angiosperm editosome [60]. The setting was different from the original study in using a bacterial expression system that allowed the simultaneous co-expression of up to 8 proteins. As a result, off-targets were detected in *E. coli*; their distribution resembles the ones detected in chloroplasts with most of the mismatches occurring in the middle of the *rpoA* target sequence. Most importantly, this new investigation tested other accessory proteins in addition of MORF2 and MORF9 and found out that ORRM1 is a genuine editing factor since its co-expression with the synthetic factor resulted in about 30% editing of the *rpoA* target.

The reliance of this first synthetic factor on the presence of accessory proteins limits its use to angiosperm plant organelles. This limitation prompted the same group responsible for the dsn3PLS-DYW to design another synthetic editor based on the tandem repeats of the more compact S-type PPR motif (Figure 8) [80]. The S-type motifs are found in plant organellar RNA editing factors and are particularly prevalent in the lycophyte Selaginella. As expected from a motif originating from a plant lacking RIP/MORF proteins, this synthetic editor shows co-factor independency and is able to edit its target in *E. coli* without requiring any additional cofactors to be added to the system. This quality combined to functionality in a wide range of pH, salt and temperature conditions make it an ideal editor to work in heterologous systems. This editor was not utilized in plant chloroplasts in this report but is very likely to be functional if given the proper transit sequence to target it to the organelle.

The proof that the DYW:KP variant domain found in seedless plants can catalyze the “reverse” editing of U to C was formally demonstrated by Ichinose et al. [37]. In their study, several DYW:KP domains based on consensus sequences extracted from hornworts, lycophytes and ferns were fused to a designer RNA-binding pentatricopeptide repeat (PPR) domain with the following motif arrangement (P1-L1-S1)3-P2-L2-S2-E1-E2-DYW (Figure 8). The PLS domain was extracted from 66 land plant genomes and designed to bind to the same *cis* element of the *rpoA* target as dsn3PLS-DYW. Three of the KP domains assayed showed U-to-C editing both in bacteria and human cells. The domain showing the strongest activity in bacteria (ca 50% U-to-C editing) has both U-to-C and C-to-U editing activities in HEK293T cells. Surprisingly, co-expression of MORF2 or MORF9 did not improve the editing efficiency of the synthetic KP editors even though their (P1-L1-S1)3-P2 motif is highly similar to dsn3PLS-DYW.

Another design strategy relied on assembling a stretch of P-type PPR consensus motif designed by Shen et al. [69] fused to the C-terminal DYW-type cytidine deaminase domain from the *P. patens* PpPPR_56 protein, including the extension motifs, E1 and E2 [81]. The 13 PPR-P motifs tract was designed to bind a 13-nt segment of the *nad7* mRNA, spanning the region from +168 to +180 relative to the start codon, with the amino acids at positions 5 and 35 of each PPR selected according to the PPR recognition code. This positioning was chosen to elicit editing of a CAA codon to a premature stop codon in the *nad7* transcript. When expressed in Arabidopsis plants, the synthetic editor achieved up to 85% editing efficiency at the target site, successfully introducing a premature stop codon in *nad7* mRNA. This resulted in reduced polysome loading of *nad7* transcripts and a phenotype characteristic of mitochondrial complex I dysfunction. This breakthrough enables sophisticated manipulation of gene expression in mitochondria, which have long been genetically inaccessible, and holds promise for applications not only in plants but also across other eukaryotes.

A very similar synthetic editor was designed by another group, with the difference being the introduction of a synthetic consensus P2–S2–L2 triplet after the last P motif and before the C-terminal E1–E2–DYW to replicate the structural arrangement found in natural PPR editing factors (Figure 8) [82]. Three different versions of the synthetic factor were designed to recognize 3 different targets, the *rpl2* and *ndhb* plastid transcripts and the mitochondrial *nad7* transcript. The editing event was planned to be silent for *rpl2* but to create premature stop codons for both *ndhB* and *nad7*. These synthetic editors elicited efficient and precise de novo RNA editing in *E. coli* as well as in the chloroplasts and mitochondria of *Nicotiana benthamiana.* With the S-type PPR-based synthetic editor, this is another editor that does not need the presence of accessory factors, introducing a new methodology in the toolbox of RNA engineering applicable to plants and potentially other organisms.

### 3.4. Other Functions than Editing for the Synthetic PPR

**Figure 9:**
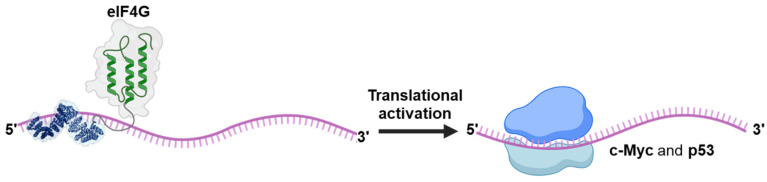
A synthetic translational activator. A P-type synPPR protein fused at its C-terminus with the translation initiation factor eIF4G effector domain enhances translation of *p53* and *c-Myc* messenger RNAs in HeLa cells.

As documented in the previous section there are several examples of synthetic editors which have been reported. It is likely that other effectors will be fused to the synthetic PPR proteins in a similar way that has occurred for the Cas 13 protein which has seen its functionality expanded by adding to its deactivated version dCas13, e.g., a demethylase [83], methyltransferase [84], peroxidase [85]. So far, only one example of a different domain than the deaminase fused to a synthetic PPR has been reported. In their study, Ping et al. [86] fused the translation initiation factor eIF4G to synthetic PPR targeting the *c-Myc* and *p53* mRNAs in mammalian cells (Figure 9). Translation activation of both mRNAs was achieved and resulted in either inhibition or stimulation of cell growth for *p53* and c-*Myc*, respectively.

## 4. Comparison with Other RNA Editors: Strengths and Limitations

### 4.1. ADAR-Based Editors

Modifying pathogenic SNPs at the RNA level has been the aim of strategies that rely on the use of fusion proteins comprising a human Adenosine Deaminase Acting on RNA (ADAR) domain with a targeting domain, which enables the interaction of the fusion protein with a guide RNA (gRNA) [87,88]. ADARs catalyze hydrolytic adenosine deamination, converting an adenosine to an inosine. As inosine is biochemically read as guanosine so that an A-to-G change is formally incorporated into the RNA, therefore potentially resulting in the reprogramming of amino acids. ADARs are naturally guided by double-strand RNA-binding domains (dsRBDs) at their N terminus that recognize and localize the enzyme to certain regions of double-stranded RNA. In the fusion proteins, the dsRBDs have been substituted by various targeting domains, a SNAP tag domain (an engineered O6-alkylguanine-DNA-alkyl transferase), a λ-phage N protein, or a catalytically dead RNA-guided Cas13b enzyme (dCas13b) [89,90,91]. The assembly of editase and gRNA mediated by these domains can be of covalent nature (SNAP) or by non-covalent interactions (λN, dCas13b). When assembled, the gRNA determines on-target specificity of the editase in a simple and programmable way, by putting the targeted adenosine into an RNA duplex, preferably into mismatch with cytidine. These different RNA editors have respective weaknesses and strengths related to the sizes and origin of the targeting domain, the sizes of the gRNAs, the nature of the bond between the gRNA and the editase, covalent or not, the delivery of the gRNA, being encodable or not. Next generation sequencing (NGS) shows a significant number of total off-target edits for all the methods, which might be problematic for therapeutic uses [91,92,93].

A cytosine deaminase for programmable C-to-U editing was engineered from the A to I deaminase domain of the hyperactive E488Q mutant of the ancestor ADAR2 [94]. With a dCas13-based RNA-targeting mechanism, the so-called RESCUE tool was able to promote specific C-to-U editing in mammalian cells. However, the RESCUE tool retained notable A-to-I off-target editing beside C-to-U off-target editing. An improved version, RESCUE-S, was developed via rational mutagenesis. The dCas13 domain of the RESCUE-S tool was replaced by a SNAP-tag for RNA-targeting [95]. This SNAP-CDAR-S was shown to outcompete the RESCUE-S tool on all tested targets; however, NGS analysis showed similar, moderate (in the hundreds) global off-target A-to-I and C-to-U editing for both tools [95].

### 4.2. Specificity and Off-Target Editing

When analyzing the specificity of the ADAR-based editors vs. the synthetic PPR-based editors, one must discriminate between the promiscuous editing occurring in the vicinity of the targeted nucleotide and editing happening more broadly elsewhere in the transcriptome. The first type of off-target editing results in the occurrence of the so-called bystander effect in a window of 5–10 nucleotides around the targeted nucleotide. This bystander effect is quite pronounced with the dCas13 based editors, presumably due to the flexibility of the linker region between the dCas13 domain and the ADAR domain, resulting in a poor spatial accuracy around the desired target [91,94]. The size of the gRNA ~85 nt comprising a 3′-terminal hairpin structure (35 nt, called DR domain) that is supposed to recruit the Cas13b fusion, and a 50 nt antisense part that binds to the target mRNA, might also favor the bystander editing of close nucleotides. The short size of gRNA (ca. 20 nt) in the SNAP-ADAR system, combined with the chemical modification of some nucleotides, suppresses the off-site editing within the gRNA-mRNA duplex [89,95]. However, in this latter system, the need for an *O*^6^-benzylguanine (BG)-moiety in the gRNA in order to bind covalently to the SNAP-ADAR makes the gRNA component not genetically encodable, which is a clear disadvantage to the system. In contrast, natural PPR editors, who do not need gRNAs, are extremely precise—editing almost always occurs at the 4th nucleotide 3′ of the nucleotide aligned with the S2 motif [42,96]. This is also the case for synthetic PPR editors; no editing has been detected at adjacent C residues [79,81,82].

Detection of off-target editing transcriptome-wide is generally achieved by deep sequencing analysis. However, several parameters could affect the readout of this analysis, such as the depth of sequencing but also the amount of the 3 components for the editing reaction, the fusion protein, the gRNA and the target RNA. As pointed out by Vogel et al., who developed the SNAP-ADAR system, the conditions under which dCas13-ADAR has been mostly characterized, namely co-transfection and overexpression of the fusion protein, the gRNA and the target, are not sufficient to support the general claims made by Cox et al., the authors of the off-target analysis study [89,91]. In particular, when overexpressed with either the wildtype ADAR2 or the SNAP-ADAR, the cas13gRNAs are similarly active as with the dCas13-ADAR, independent of the DR domain responsible for the recruitment of dCas13 [89]. This shows that any overexpressed highly active ADAR can edit 50 bp gRNA/mRNA duplexes independent of a targeting mechanism. Furthermore, for their deep sequencing analysis, Cox et al. used 15-fold less plasmid encoding the Cas-ADAR than in the editing reactions. Again, as pointed out by Vogel et al., one can expect a significant reduction in transfection efficiency by lowering the amount of Cas-ADAR, thus artificially reducing the global off-target editing by diluting the off-target with many untransfected cells, while the editing of the co-transfected reporter transcript is less affected.

A more rigorous setting was used to compare the off-target editing caused by the SNAP-CDAR-S and Cas13-RESCUE-S, which were both stably integrated as a single copy in the genome of mammalian cells [95]. Artifacts in off-target analyses caused by overexpression of the editases were thus eliminated. Comparisons were made between cells expressing the respective editing effectors in the presence and absence of the respective guide RNA and cells not expressing an engineered effector. The patterns between the two effectors were very similar, which was expected given that the ADAR domain of both editing tools is identical. More importantly, the vast majority of off-target editing came from the presence of the editing enzymes, was guide RNA-independent and amounted in the thousands for A-to-I and hundreds for C-to-U for both editases. Over a hundred of missense mutations occurred, which limit the therapeutic use of these editors.

Most of the synPPR editors have been tested either in bacteria or in the plant organelles [79,80,81,82], which possess transcriptomes order of magnitude less complex than mammalian transcriptomes. It is therefore difficult to establish a comparison with ADAR-based editors. The only synPPR editor tested in mammalian cells carried the DYW:KP domain responsible for U-to-C editing and created 98 U-to-C editing off-target sites in HEK293T cells [37]. A direct comparison between the synPPR editors and the ADAR-based editors is lacking regarding their off-target performances. A useful design would deliver both tools to the same cell type against the same RNA target and quantify specificity and efficiency by high throughput RNA sequencing. An RNA-binding study of synPPR-P protein showed that proteins with 11 and 14 repeats exhibit similar affinity for their intended targets but 14-repeats are more permissive for mismatches [23]. Therefore, there is a limit to the length of the PPR protein over which increasing the number of repeats does not increase the affinity for the RNA ligand but instead favors the occurrence of off-target editing. Given the one PPR/one ribonucleotide model of recognition, a 11-repeat synPPR would target a 11-nucleotide RNA sequence. There are 4^11^ or 4 million possible variations of this sequence. Therefore, such a discriminatory power is likely to be efficient in the context of an organellar genome but unlikely to work in the context of a more complex nuclear genome. A possible solution to increase the discriminatory power of synPPR editors would be to split the editing domain and to tether the two halves to two different synPPR proteins, each one recognizing 11 ribonucleotides upstream and downstream the targeted nucleotide [97]. This architecture is similar to the one used in TALEN (Transcription Activator-Like Effector Nuclease) where two TALEN proteins must bind to opposite DNA strands, and their FokI domains must then come together (dimerize) to create a double-strand break.

### 4.3. Context Dependency for Efficient Editing

ADAR-based RNA editors can perform C-to-U [94,95] and A-to-I conversion [89,91] while synPPR editors convert C-to-U [79,80] and U-to-C [37] on RNA transcripts. While transitions are the most common pathogenic SNP (47% of C > T/G > A, 21% of A > G/T > C, estimates based on ClinVar), there are other constraints to be reckoned with when considering these editors to correct deleterious mutations. Analysis of off-target editing by synPPR editors show a marked bias toward pyrimidine at position −1 directly upstream of the targeted nucleotide [37,60,81]. It has been shown previously that a guanine at position −1 on the RNA target has a negative impact on editing efficiency by almost systematically obliterating it [33,98,99,100]. There is a similar neighbor preference for the ADAR enzyme with guanine being the less favored nucleotide directly upstream of the adenine targeted for deamination. The same bias is observed with the fusion editase SNAP-ADAR where editing efficiency is poor for GAN triplets [89]. This defect was also present in the SNAP-CDAR-S where the editase is an evolved ADAR deaminase preferentially deaminating the cytidine [95].

## 5. Conclusions

The recent development of synthetic PPR proteins and editors has transformed a once enigmatic plant-specific phenomenon into a versatile molecular platform for programmable RNA manipulation. In just a few years, the field has progressed from deciphering the PPR code to demonstrating efficient, site-specific RNA editing in heterologous systems ranging from bacteria to human cells. These advances underscore the potential of PPR-based tools to complement and extend existing programmable RNA-binding systems such as PUFs and Cas13 derivatives.

Yet the full potential of synthetic PPR editors is far from realized. Several conceptual and technical challenges remain. First, although the modular PPR code provides a predictable basis for target recognition, binding specificity is not absolute. Mismatches, off-target binding, and the influence of local RNA structure can significantly affect performance, particularly in the complex cytoplasmic transcriptomes of non-plant cells. Refining the design rules that govern sequence discrimination, motif length, and tolerance to secondary structures will be crucial to expand the precision and scope of PPR-based targeting.

Secondly, the modular nature of the DYW catalytic domain—originally adapted for plant organellar environments—poses questions about its compatibility with diverse intracellular contexts. Systematic exploration of natural DYW variants, and even directed evolution of their catalytic cores, may yield editors with a broader substrate range and improved biochemical robustness.

Thirdly, expanding the functional repertoire of PPR-based systems offers an especially promising direction. Beyond cytidine and uridine deamination, synthetic PPRs could be fused to effectors mediating adenosine deamination, methylation, demethylation, or RNA cleavage, thereby creating a comprehensive “toolkit” for RNA-level engineering. The successful fusion of translational activators to synthetic PPRs in mammalian cells already demonstrates the feasibility of coupling modular RNA recognition to orthogonal effector outputs.

Finally, PPR editors hold promise not only as molecular tools but also as probes of fundamental RNA biology. Their predictable architecture and tunable specificity make them ideal scaffolds for dissecting RNA processing, translation, and organellar gene regulation in vivo. In therapeutic contexts, the demonstration of PPR-mediated correction of RNA toxicity in a mouse model of myotonic dystrophy underscores their translational potential. Continued improvements in delivery systems, expression stability, and catalytic efficiency will be key to extending these applications to human disease.

In summary, synthetic PPR editors are emerging as a unique class of programmable RNA effectors distinguished by their modularity, composability, and evolutionary versatility. The next phase of research will likely focus on (i) refining targeting predictability and off-target assessment, and (ii) integrating PPR scaffolds with synthetic biology frameworks for dynamic control of RNA function. Together, these advances will move synthetic PPR proteins from proof-of-concept tools to generalizable platforms for precision RNA engineering across the tree of life.

## Data Availability

No new data were created or analyzed in this study. Data sharing is not applicable to this article.

## Abbreviations

The following abbreviations are used in this manuscript:

PPR	Pentatricopeptide Repeat
synPPR	Synthetic PPR
RIP/MORF	RNA editing factor Interacting Protein/Multiple Organellar RNA editing Factor
ORRM	Organelle RNA Recognition Motif

## References

[1] Gott J.M., Emeson R.B. (2000). Functions and mechanisms of RNA editing. Annu. Rev. Genet..

[2] Covello P.S., Gray M.W. (1989). RNA editing in plant mitochondria. Nature.

[3] Gualberto J.M., Lamattina L., Bonnard G., Weil J.H., Grienenberger J.M. (1989). RNA editing in wheat mitochondria results in the conservation of protein sequences. Nature.

[4] Freyer R., Kiefer-Meyer M.C., Kossel H. (1997). Occurrence of plastid RNA editing in all major lineages of land plants. Proc. Natl. Acad. Sci. USA.

[5] Hermann M., Bock R. (1999). Transfer of plastid RNA-editing activity to novel sites suggests a critical role for spacing in editing-site recognition. Proc. Natl. Acad. Sci. USA.

[6] Smith H.C., Gott J.M., Hanson M.R. (1997). A guide to RNA editing. RNA.

[7] Knie N., Grewe F., Fischer S., Knoop V. (2016). Reverse U-to-C editing exceeds C-to-U RNA editing in some ferns—A monilophyte-wide comparison of chloroplast and mitochondrial RNA editing suggests independent evolution of the two processes in both organelles. BMC Evol. Biol..

[8] Gerke P., Szovenyi P., Neubauer A., Lenz H., Gutmann B., McDowell R., Small I., Schallenberg-Rudinger M., Knoop V. (2020). Towards a plant model for enigmatic U-to-C RNA editing: The organelle genomes, transcriptomes, editomes and candidate RNA editing factors in the hornwort *Anthoceros agrestis*. New Phytol..

[9] Kudla J., Igloi G.L., Metzlaff M., Hagemann R., Kossel H. (1992). RNA editing in tobacco chloroplasts leads to the formation of a translatable psbL mRNA by a C to U substitution within the initiation codon. EMBO J..

[10] Wakasugi T., Hirose T., Horihata M., Tsudzuki T., Kossel H., Sugiura M. (1996). Creation of a novel protein-coding region at the RNA level in black pine chloroplasts: The pattern of RNA editing in the gymnosperm chloroplast is different from that in angiosperms. Proc. Natl. Acad. Sci. USA.

[11] Kotera E., Tasaka M., Shikanai T. (2005). A pentatricopeptide repeat protein is essential for RNA editing in chloroplasts. Nature.

[12] Hammani K., Okuda K., Tanz S.K., Chateigner-Boutin A.L., Shikanai T., Small I. (2009). A study of new Arabidopsis chloroplast RNA editing mutants reveals general features of editing factors and their target sites. Plant Cell.

[13] Zehrmann A., Verbitskiy D., van der Merwe J.A., Brennicke A., Takenaka M. (2009). A DYW domain-containing pentatricopeptide repeat protein is required for RNA editing at multiple sites in mitochondria of *Arabidopsis thaliana*. Plant Cell.

[14] Chaudhuri S., Maliga P. (1996). Sequences directing C to U editing of the plastid *psbL* mRNA are located within a 22 nucleotide segment spanning the editing site. EMBO J..

[15] Hirose T., Sugiura M. (2001). Involvement of a site-specific *trans*-acting factor and a common RNA-binding protein in the editing of chloroplast mRNAs: Development of a chloroplast in vitro RNA editing system. EMBO J..

[16] Barkan A., Small I. (2014). Pentatricopeptide repeat proteins in plants. Annu. Rev. Plant Biol..

[17] Wang M., Oge L., Perez-Garcia M.D., Hamama L., Sakr S. (2018). The PUF Protein Family: Overview on PUF RNA Targets, Biological Functions, and Post Transcriptional Regulation. Int. J. Mol. Sci..

[18] Robles P., Micol J.L., Quesada V. (2012). Unveiling plant mTERF functions. Mol. Plant.

[19] Lefebvre-Legendre L., Choquet Y., Kuras R., Loubery S., Douchi D., Goldschmidt-Clermont M. (2015). A nucleus-encoded chloroplast protein regulated by iron availability governs expression of the photosystem I subunit *PsaA* in *Chlamydomonas reinhardtii*. Plant Physiol..

[20] Hillebrand A., Matz J.M., Almendinger M., Muller K., Matuschewski K., Schmitz-Linneweber C. (2018). Identification of clustered organellar short (cos) RNAs and of a conserved family of organellar RNA-binding proteins, the heptatricopeptide repeat proteins, in the malaria parasite. Nucleic Acids Res..

[21] Preker P.J., Keller W. (1998). The HAT helix, a repetitive motif implicated in RNA processing. Trends Biochem. Sci..

[22] Liu S., Melonek J., Boykin L.M., Small I., Howell K.A. (2013). PPR-SMRs: Ancient proteins with enigmatic functions. RNA Biol..

[23] Miranda R.G., McDermott J.J., Barkan A. (2018). RNA-binding specificity landscapes of designer pentatricopeptide repeat proteins elucidate principles of PPR-RNA interactions. Nucleic Acids Res..

[24] O’Toole N., Hattori M., Andres C., Iida K., Lurin C., Schmitz-Linneweber C., Sugita M., Small I. (2008). On the expansion of the pentatricopeptide repeat gene family in plants. Mol. Biol. Evol..

[25] Lurin C., Andres C., Aubourg S., Bellaoui M., Bitton F., Bruyere C., Caboche M., Debast C., Gualberto J., Hoffmann B. (2004). Genome-wide analysis of Arabidopsis pentatricopeptide repeat proteins reveals their essential role in organelle biogenesis. Plant Cell.

[26] Small I.D., Peeters N. (2000). The PPR motif—A TPR-related motif prevalent in plant organellar proteins. Trends Biochem. Sci..

[27] Gobert A., Gutmann B., Taschner A., Gossringer M., Holzmann J., Hartmann R.K., Rossmanith W., Giege P. (2010). A single Arabidopsis organellar protein has RNase P activity. Nat. Struct. Mol. Biol..

[28] Puchta O., Lubas M., Lipinski K.A., Piatkowski J., Malecki M., Golik P. (2010). DMR1 (CCM1/YGR150C) of *Saccharomyces cerevisiae* encodes an RNA-binding protein from the pentatricopeptide repeat family required for the maintenance of the mitochondrial 15S ribosomal RNA. Genetics.

[29] Beick S., Schmitz-Linneweber C., Williams-Carrier R., Jensen B., Barkan A. (2008). The pentatricopeptide repeat protein PPR5 stabilizes a specific tRNA precursor in maize chloroplasts. Mol. Cell. Biol..

[30] Prikryl J., Rojas M., Schuster G., Barkan A. (2011). Mechanism of RNA stabilization and translational activation by a pentatricopeptide repeat protein. Proc. Natl. Acad. Sci. USA.

[31] Zhou W., Lu Q., Li Q., Wang L., Ding S., Zhang A., Wen X., Zhang L., Lu C. (2017). PPR-SMR protein SOT1 has RNA endonuclease activity. Proc. Natl. Acad. Sci. USA.

[32] Huynh S.D., Melonek J., Colas des Francs-Small C., Bond C.S., Small I. (2023). A unique C-terminal domain contributes to the molecular function of Restorer-of-fertility proteins in plant mitochondria. New Phytol..

[33] Oldenkott B., Yang Y., Lesch E., Knoop V., Schallenberg-Rudinger M. (2019). Plant-type pentatricopeptide repeat proteins with a DYW domain drive C-to-U RNA editing in *Escherichia coli*. Commun. Biol..

[34] Okuda K., Myouga F., Motohashi R., Shinozaki K., Shikanai T. (2007). Conserved domain structure of pentatricopeptide repeat proteins involved in chloroplast RNA editing. Proc. Natl. Acad. Sci. USA.

[35] Okuda K., Chateigner-Boutin A.L., Nakamura T., Delannoy E., Sugita M., Myouga F., Motohashi R., Shinozaki K., Small I., Shikanai T. (2009). Pentatricopeptide repeat proteins with the DYW motif have distinct molecular functions in RNA editing and RNA cleavage in Arabidopsis chloroplasts. Plant Cell.

[36] Rudinger M., Polsakiewicz M., Knoop V. (2008). Organellar RNA editing and plant-specific extensions of pentatricopeptide repeat proteins in jungermanniid but not in marchantiid liverworts. Mol. Biol. Evol..

[37] Ichinose M., Kawabata M., Akaiwa Y., Shimajiri Y., Nakamura I., Tamai T., Nakamura T., Yagi Y., Gutmann B. (2022). U-to-C RNA editing by synthetic PPR-DYW proteins in bacteria and human culture cells. Commun. Biol..

[38] Okuda K., Nakamura T., Sugita M., Shimizu T., Shikanai T. (2006). Apentatricopeptide repeat protein is a site recognition factor in chloroplast RNA editing. J. Biol. Chem..

[39] Yin P., Li Q., Yan C., Liu Y., Liu J., Yu F., Wang Z., Long J., He J., Wang H.W. (2013). Structural basis for the modular recognition of single-stranded RNA by PPR proteins. Nature.

[40] Fujii S., Bond C.S., Small I.D. (2011). Selection patterns on restorer-like genes reveal a conflict between nuclear and mitochondrial genomes throughout angiosperm evolution. Proc. Natl. Acad. Sci. USA.

[41] Barkan A., Rojas M., Fujii S., Yap A., Chong Y.S., Bond C.S., Small I. (2012). A combinatorial amino acid code for RNA recognition by pentatricopeptide repeat proteins. PLoS Genet..

[42] Yagi Y., Hayashi S., Kobayashi K., Hirayama T., Nakamura T. (2013). Elucidation of the RNA recognition code for pentatricopeptide repeat proteins involved in organelle RNA editing in plants. PLoS ONE.

[43] Kindgren P., Yap A., Bond C.S., Small I. (2015). Predictable alteration of sequence recognition by RNA editing factors from Arabidopsis. Plant Cell.

[44] McDermott J.J., Watkins K.P., Williams-Carrier R., Barkan A. (2019). Ribonucleoprotein capture by in vivo expression of a designer pentatricopeptide repeat protein in Arabidopsis. Plant Cell.

[45] Salone V., Rudinger M., Polsakiewicz M., Hoffmann B., Groth-Malonek M., Szurek B., Small I., Knoop V., Lurin C. (2007). A hypothesis on the identification of the editing enzyme in plant organelles. FEBS Lett..

[46] Boussardon C., Salone V., Avon A., Berthome R., Hammani K., Okuda K., Shikanai T., Small I., Lurin C. (2012). Two interacting proteins are necessary for the editing of the *NdhD*-1 site in Arabidopsis plastids. Plant Cell.

[47] Andres-Colas N., Zhu Q., Takenaka M., De Rybel B., Weijers D., Van Der Straeten D. (2017). Multiple PPR protein interactions are involved in the RNA editing system in Arabidopsis mitochondria and plastids. Proc. Natl. Acad. Sci. USA.

[48] Hayes M.L., Dang K.N., Diaz M.F., Mulligan R.M. (2015). A conserved glutamate residue in the C-terminal deaminase domain of pentatricopeptide repeat proteins is required for RNA editing activity. J. Biol. Chem..

[49] Wagoner J.A., Sun T., Lin L., Hanson M.R. (2015). Cytidine deaminase motifs within the DYW domain of two pentatricopeptide repeat-containing proteins are required for site-specific chloroplast RNA editing. J. Biol. Chem..

[50] Hayes M.L., Santibanez P.I. (2020). A plant pentatricopeptide repeat protein with a DYW-deaminase domain is sufficient for catalyzing C-to-U RNA editing in vitro. J. Biol. Chem..

[51] Bentolila S., Heller W.P., Sun T., Babina A.M., Friso G., van Wijk K.J., Hanson M.R. (2012). RIP1, a member of an Arabidopsis protein family, interacts with the protein RARE1 and broadly affects RNA editing. Proc. Natl. Acad. Sci. USA.

[52] Takenaka M., Zehrmann A., Verbitskiy D., Kugelmann M., Hartel B., Brennicke A. (2012). Multiple organellar RNA editing factor (MORF) family proteins are required for RNA editing in mitochondria and plastids of plants. Proc. Natl. Acad. Sci. USA.

[53] Bentolila S., Oh J., Hanson M.R., Bukowski R. (2013). Comprehensive high-resolution analysis of the role of an Arabidopsis gene family in RNA editing. PLoS Genet..

[54] Glass F., Hartel B., Zehrmann A., Verbitskiy D., Takenaka M. (2015). MEF13 requires MORF3 and MORF8 for RNA editing at eight targets in mitochondrial mRNAs in *Arabidopsis thaliana*. Mol. Plant.

[55] Yan J., Zhang Q., Guan Z., Wang Q., Li L., Ruan F., Lin R., Zou T., Yin P. (2017). MORF9 increases the RNA-binding activity of PLS-type pentatricopeptide repeat protein in plastid RNA editing. Nat. Plants.

[56] Sun T., Germain A., Giloteaux L., Hammani K., Barkan A., Hanson M.R., Bentolila S. (2013). An RNA recognition motif-containing protein is required for plastid RNA editing in Arabidopsis and maize. Proc. Natl. Acad. Sci. USA.

[57] Shi X., Hanson M.R., Bentolila S. (2015). Two RNA recognition motif-containing proteins are plant mitochondrial editing factors. Nucleic Acids Res..

[58] Shi X., Germain A., Hanson M.R., Bentolila S. (2016). RNA Recognition Motif-containing protein ORRM4 broadly affects mitochondrial RNA editing and impacts plant development and flowering. Plant Physiol..

[59] Hackett J.B., Shi X., Kobylarz A.T., Lucas M.K., Wessendorf R.L., Hines K.M., Bentolila S., Hanson M.R., Lu Y. (2017). An organelle RNA Recognition Motif protein is required for photosystem II subunit *psbF* transcript editing. Plant Physiol..

[60] Lombana J.M., Hanson M.R., Bentolila S. (2025). Deciphering the role of accessory proteins in Arabidopsis chloroplast editosomes via interaction with a synthetic PPR-PLS factor in *E. coli*. Nucleic Acids Res..

[61] Zhang F., Tang W., Hedtke B., Zhong L., Liu L., Peng L., Lu C., Grimm B., Lin R. (2014). Tetrapyrrole biosynthetic enzyme protoporphyrinogen IX oxidase 1 is required for plastid RNA editing. Proc. Natl. Acad. Sci. USA.

[62] Bobik K., McCray T.N., Ernest B., Fernandez J.C., Howell K.A., Lane T., Staton M., Burch-Smith T.M. (2017). The chloroplast RNA helicase ISE2 is required for multiple chloroplast RNA processing steps in Arabidopsis thaliana. Plant J..

[63] Sun T., Shi X., Friso G., Van Wijk K., Bentolila S., Hanson M.R. (2015). A zinc finger motif-containing protein is essential for chloroplast RNA editing. PLoS Genet..

[64] Bentolila S., Gipson A.B., Kehl A.J., Hamm L.N., Hayes M.L., Mulligan R.M., Hanson M.R. (2021). A RanBP2-type zinc finger protein functions in intron splicing in Arabidopsis mitochondria and is involved in the biogenesis of respiratory complex I. Nucleic Acids Res..

[65] Sandoval R., Boyd R.D., Kiszter A.N., Mirzakhanyan Y., Santibanez P., Gershon P.D., Hayes M.L. (2019). Stable native RIP9 complexes associate with C-to-U RNA editing activity, PPRs, RIPs, OZ1, ORRM1 and ISE2. Plant J..

[66] Guillaumot D., Lopez-Obando M., Baudry K., Avon A., Rigaill G., Falcon de Longevialle A., Broche B., Takenaka M., Berthome R., De Jaeger G. (2017). Two interacting PPR proteins are major Arabidopsis editing factors in plastid and mitochondria. Proc. Natl. Acad. Sci. USA.

[67] Sun T., Bentolila S., Hanson M.R. (2016). The unexpected diversity of plant organelle RNA editosomes. Trends Plant Sci..

[68] Gully B.S., Shah K.R., Lee M., Shearston K., Smith N.M., Sadowska A., Blythe A.J., Bernath-Levin K., Stanley W.A., Small I.D. (2015). The design and structural characterization of a synthetic pentatricopeptide repeat protein. Acta Crystallogr. D Biol. Crystallogr..

[69] Shen C., Wang X., Liu Y., Li Q., Yang Z., Yan N., Zou T., Yin P. (2015). Specific RNA recognition by designer pentatricopeptide repeat protein. Mol. Plant.

[70] Coquille S., Filipovska A., Chia T., Rajappa L., Lingford J.P., Razif M.F., Thore S., Rackham O. (2014). An artificial PPR scaffold for programmable RNA recognition. Nat. Commun..

[71] Johnson X., Wostrikoff K., Finazzi G., Kuras R., Schwarz C., Bujaldon S., Nickelsen J., Stern D.B., Wollman F.A., Vallon O. (2010). MRL1, a conserved Pentatricopeptide repeat protein, is required for stabilization of *rbcL* mRNA in Chlamydomonas and Arabidopsis. Plant Cell.

[72] Manavski N., Mathieu S., Rojas M., Meteignier L.V., Brachmann A., Barkan A., Hammani K. (2021). In vivo stabilization of endogenous chloroplast RNAs by customized artificial pentatricopeptide repeat proteins. Nucleic Acids Res..

[73] Rojas M., Chotewutmontri P., Barkan A. (2024). Translational activation by a synthetic PPR protein elucidates control of *psbA* translation in Arabidopsis chloroplasts. Plant Cell.

[74] Manavski N., Schwenkert S., Kunz H.H., Leister D., Meurer J. (2025). Targeted translation inhibition of chloroplast and mitochondrial mRNAs by designer pentatricopeptide repeat proteins. Nucleic Acids Res..

[75] Marzano N., Johnston B., Paudel B.P., Schmidberger J., Jergic S., Bocking T., Agostino M., Small I., van Oijen A.M., Bond C.S. (2024). Single-molecule visualization of sequence-specific RNA binding by a designer PPR protein. Nucleic Acids Res..

[76] Yagi Y., Teramoto T., Kaieda S., Imai T., Sasaki T., Yagi M., Maekawa N., Nakamura T. (2022). Construction of a versatile, programmable RNA-binding protein using designer PPR proteins and its application for splicing control in mammalian cells. Cells.

[77] Imai T., Miyai M., Nemoto J., Tamai T., Ohta M., Yagi Y., Nakanishi O., Mochizuki H., Nakamori M. (2025). Pentatricopeptide repeat protein targeting CUG repeat RNA ameliorates RNA toxicity in a myotonic dystrophy type 1 mouse model. Sci. Transl. Med..

[78] Spahr H., Chia T., Lingford J.P., Siira S.J., Cohen S.B., Filipovska A., Rackham O. (2018). Modular ssDNA binding and inhibition of telomerase activity by designer PPR proteins. Nat. Commun..

[79] Royan S., Gutmann B., Colas des Francs-Small C., Honkanen S., Schmidberger J., Soet A., Sun Y.K., Vincis Pereira Sanglard L., Bond C.S., Small I. (2021). A synthetic RNA editing factor edits its target site in chloroplasts and bacteria. Commun. Biol..

[80] Bernath-Levin K., Schmidberger J., Honkanen S., Gutmann B., Sun Y.K., Pullakhandam A., Colas des Francs-Small C., Bond C.S., Small I. (2021). Cofactor-independent RNA editing by a synthetic S-type PPR protein. Synth. Biol..

[81] Manavski N., Abdel-Salam E., Schwenkert S., Kunz H.H., Brachmann A., Leister D., Meurer J. (2025). Targeted introduction of premature stop codon in plant mitochondrial mRNA by a designer pentatricopeptide repeat protein with C-to-U editing function. Plant J..

[82] Mathieu S., Lesch E., Garcia S., Graindorge S., Schallenberg-Rudinger M., Hammani K. (2025). De novo RNA base editing in plant organelles with engineered synthetic P-type PPR editing factors. Nucleic Acids Res..

[83] Li J., Chen Z., Chen F., Xie G., Ling Y., Peng Y., Lin Y., Luo N., Chiang C.M., Wang H. (2020). Targeted mRNA demethylation using an engineered dCas13b-ALKBH5 fusion protein. Nucleic Acids Res..

[84] Wilson C., Chen P.J., Miao Z., Liu D.R. (2020). Programmable m^6^A modification of cellular RNAs with a Cas13-directed methyltransferase. Nat. Biotechnol..

[85] Han S., Zhao B.S., Myers S.A., Carr S.A., He C., Ting A.Y. (2020). RNA-protein interaction mapping via MS2- or Cas13-based APEX targeting. Proc. Natl. Acad. Sci. USA.

[86] Ping N., Hara-Kuge S., Yagi Y., Kazama T., Nakamura T. (2024). Translational enhancement of target endogenous mRNA in mammalian cells using programmable RNA-binding pentatricopeptide repeat proteins. Sci. Rep..

[87] Rees H.A., Liu D.R. (2018). Base editing: Precision chemistry on the genome and transcriptome of living cells. Nat. Rev. Genet..

[88] Vogel P., Stafforst T. (2019). Critical review on engineering deaminases for site-directed RNA editing. Curr. Opin. Biotechnol..

[89] Vogel P., Moschref M., Li Q., Merkle T., Selvasaravanan K.D., Li J.B., Stafforst T. (2018). Efficient and precise editing of endogenous transcripts with SNAP-tagged ADARs. Nat. Methods.

[90] Montiel-Gonzalez M.F., Vallecillo-Viejo I., Yudowski G.A., Rosenthal J.J. (2013). Correction of mutations within the cystic fibrosis transmembrane conductance regulator by site-directed RNA editing. Proc. Natl. Acad. Sci. USA.

[91] Cox D.B.T., Gootenberg J.S., Abudayyeh O.O., Franklin B., Kellner M.J., Joung J., Zhang F. (2017). RNA editing with CRISPR-Cas13. Science.

[92] Stroppel A.S., Latifi N., Hanswillemenke A., Tasakis R.N., Papavasiliou F.N., Stafforst T. (2021). Harnessing self-labeling enzymes for selective and concurrent A-to-I and C-to-U RNA base editing. Nucleic Acids Res..

[93] Vallecillo-Viejo I.C., Liscovitch-Brauer N., Montiel-Gonzalez M.F., Eisenberg E., Rosenthal J.J.C. (2018). Abundant off-target edits from site-directed RNA editing can be reduced by nuclear localization of the editing enzyme. RNA Biol..

[94] Abudayyeh O.O., Gootenberg J.S., Franklin B., Koob J., Kellner M.J., Ladha A., Joung J., Kirchgatterer P., Cox D.B.T., Zhang F. (2019). A cytosine deaminase for programmable single-base RNA editing. Science.

[95] Latifi N., Mack A.M., Tellioglu I., Di Giorgio S., Stafforst T. (2023). Precise and efficient C-to-U RNA base editing with SNAP-CDAR-S. Nucleic Acids Res..

[96] Kobayashi T., Yagi Y., Nakamura T. (2019). Comprehensive prediction of target RNA editing sites for PLS-class PPR proteins in *Arabidopsis thaliana*. Plant Cell Physiol..

[97] McDowell R., Small I., Bond C.S. (2022). Synthetic PPR proteins as tools for sequence-specific targeting of RNA. Methods.

[98] Miyamoto T., Obokata J., Sugiura M. (2004). A site-specific factor interacts directly with its cognate RNA editing site in chloroplast transcripts. Proc. Natl. Acad. Sci. USA.

[99] Choury D., Farre J.C., Jordana X., Araya A. (2004). Different patterns in the recognition of editing sites in plant mitochondria. Nucleic Acids Res..

[100] Yang Y., Ritzenhofen K., Otrzonsek J., Xie J., Schallenberg-Rudinger M., Knoop V. (2023). Beyond a PPR-RNA recognition code: Many aspects matter for the multi-targeting properties of RNA editing factor PPR56. PLoS Genet..

